# Adjuvant Pelvic Radiotherapy *vs.* Sequential Chemoradiotherapy for High-Risk Stage I-II Endometrial Carcinoma

**DOI:** 10.7497/j.issn.2095-3941.2012.03.003

**Published:** 2012-09

**Authors:** Hend Ahmed El-Hadaad, Hanan Ahmed Wahba, Anas Mohamed Gamal, Tamer Dawod

**Affiliations:** 1 Department of Clinical Oncology and Nuclear Medicine,; 2 Gynecology and Obstetrics,; 3 Medical Physics, Mansoura University, Mansoura 35516, Egypt

**Keywords:** stage I-II high-risk endometrial cancer, adjuvant radiotherapy, adjuvant sequential chemoradiotherapy

## Abstract

**Objective:**

To explore if the addition of adjuvant chemotherapy with paclitaxel and carboplatin to radiotherapy confers an advantage for overall survival (OAS), and progression free survival (PFS); to assess the incidence of relapses over standard pelvic radiotherapy; and to evaluate the related toxicity in high-risk stage I-II endometrial carcinoma

**Methods:**

Medical records were reviewed to identify high-risk stage I-II endometrial carcinoma cases treated in the Clinical Oncology and Nuclear Medicine department between 2002 and 2008 with adjuvant radiotherapy alone (arm I) (57 patients) or with sequential carboplatin (AUC5-6) and paclitaxel (135−175 mg/m^2^) with radiotherapy (arm II) (51 patients). Radiotherapy was performed through the four-field box technique at doses of 45−50 Gy (1.8 Gy/day × 5 days/week).

**Results:**

The toxicity was manageable and predominantly hematologic with a grade 3 neutropenia and thrombocytopenia in 9.8% and 6% of the patients in arm I and arm II, respectively, without febrile neutropenia. All patients experienced hair loss. Chemoradiotherapy arm was associated with a lower incidence rate of relapse (9.8% *vs.* 22.7%). After a median follow-up period of 48 months, the 5-year OAS and PFS rates for chemoradiotherapy-treated patients were significantly more favorable than those who did not receive chemotherapy (*P*=0.02 and 0.03, respectively). In arm I, the OAS and PFS rates were 73.7% and 66.7% compared with those in arm II, whose rates were 90.2% and 84.3%.

**Conclusions:**

Adjuvant chemoradiation with paclitaxel and carboplatin improved the survival rates and decreased the recurrence rates in patients with high-risk stage I-II endometrial carcinoma. Chemotherapy was associated with an acceptable rate of toxicity. However, a prospective study with a larger number of patients is needed to define a standard adjuvant treatment for high-risk stage I-II endometrial carcinoma.

## Introduction

Endometrial cancer is the most common gynecological malignancy in Europe and North America, and is the seventh most common cause of death from cancer among women in Western Europe^[^^1^^]^.

The International Federation of Gynecology and Obstetrics (FIGO) considered the surgical stage as the most important independent prognostic indicator of OAS or PFS, and this stage has a significant impact on treatment decisions^[^^2^^]^. In the early stages (stage I-II) of endometrial cancer, several independent prognostic factors were identified such as lymph vascular space involvement, histologic grade 3, aggressive pathologic subtypes (uterine papillary serous carcinoma and clear cell carcinoma), deep myometrial invasion, cervical invasion, and age above 60 years^[^^3^^]^. Patients exhibiting any of these features are often characterized to be at high risk and adjuvant therapy is often recommended. Prior clinical trials using adjuvant radiotherapy has shown a reduced risk of local relapses. However, adjuvant radiotherapy did not improve overall survival because of the development of distant metastases^[^^4^^,^^5^^]^.

Jolly et al.^[^^6^^]^ reported that vaginal brachytherapy alone yielded similar overall survival and cumulative recurrence rates to the standard external pelvic radiotherapy in stage I-II endometrial cancer. Chemotherapy trials have demonstrated that the combination of adriamycin and cisplatin is more effective than single agent therapy^[^^7^^,^^8^^]^.

Substitution of carboplatin for cisplatin may improve tolerability without sacrificing efficacy. Myelosuppression may be more frequent, but nephrotoxicity, neurotoxicity, and emesis were all less frequently reported and were milder with carboplatin than those of cisplatin-based regimen^[^^9^^]^.

Other chemotherapeutic agents such as paclitaxel have shown promising survival and response rates in endometrial cancer. Paclitaxel has been associated with favorable rates either as a single agent or in combination with platinum-based chemotherapy^[^^10^^]^.

Given the risks of both local and distant relapses, the combined chemotherapy and radiation either concomitantly or sequentially has gained increasing attention. Duke et al. reported their findings based on a large retrospective study of chemotherapy *vs.* radiation *vs.* concomitant chemoradiation among women with stage III or IV uterine cancer^[^^11^^]^. Their analysis suggested that chemoradiation improved both the progression free survival and overall survival. Therefore, we conducted this study based on the following objectives: to explore if the addition of adjuvant chemotherapy with paclitaxel and carboplatin to radiotherapy confers an advantage for overall survival and progression free survival; to assess the incidence of relapses over standard pelvic radiotherapy; and to evaluate the related toxicity in high-risk stage I-II endometrial cancer.

## Patients and Methods

### Patients

This retrospective study included 108 patients with endometrial cancer presented to the Department of Clinical Oncology and Nuclear Medicine, Mansoura University Hospital between January 2002 and December 2008. Demographic and treatment data were collected by checking through the patient’s files for the date and pattern of progression, date of death or last follow-up, and the incidences of toxicities.

### Eligible criteria

All patients underwent total abdominal hysterectomy with bilateral salpingo-oophrectomy (TAH-BSO) with no residual disease. No routine pelvic lymphadenectomy was done, and only sampling was performed to examine any suspicious lymph node. Other eligible criteria were: *i*) non-metastatic patients with histologically confirmed FIGO (2009)^[^^12^^]^ stage I-II endometrial cancer (IA tumor confined to the uterus, without or <1/2 myometrial invasion, IB Tumor confined to the uterus, ≥1/2 myometrial invasion, II cervical stromal invasion but not beyond the uterus) with one or more of the following risk factors: lymphovascular invasion, histologic grade 3, aggressive pathology (papillary serous and clear cell carcinoma), and age above 60 years; *ii*) Patients who received adjuvant pelvic radiotherapy (RT) (arm I) or chemoradiotherapy (arm II).

Arm I included 57 patients who received pelvic RT at doses of 45-50 Gy (1.8 Gy/d × 5 days/week) through the 4-field box technique. The upper border of the pelvic field was at L4-L5; the lower border was at the lower border of the vagina indicated by a marker; the lateral border was at 1.5 cm lateral to the pelvic brim; posteriorly at S3; and anteriorly at the symphysis pubis.

Arm II included 51 patients treated by 4 cycles of paclitaxel at doses of 135-175 mg/m^2^ and carboplatin AUC 5-6 at 3-week intervals following the completion of pelvic RT. All patients completed 4 cycles of chemotherapy based on their treatment sheets.

The primary end points were progression free survival (PFS), which was calculated from the date of diagnosis to the date of progression, and overall survival (OAS) rate, which was calculated from the date of diagnosis to the date of patient’s death or to the date of last follow-up. The secondary end point was treatment-related toxicities.

Systemic toxicity of chemotherapy was graded in accordance with the National Cancer Institute Common Toxicity Criteria (NCI-CTC) version 3.0^[^^13^^]^.

### Statistical analysis

The data were encoded in a computer using the Statistical Package for Social Sciences (SPSS) version 15.0 (Chicago, IL, USA)^[^^14^^]^. The results were expressed as numbers, percentages, and medians because the data were non-normal distributions. Categorical variables were compared using the Fisher test. The survival functions (OAS and PFS) were estimated using the Kaplan Meier test. The Log rank test was used to analyze the differences between the curves. All statistical tests were two-sided with a *P*-value of <0.05 as statistically significant.

## Results

We identified 108 women who met the eligibility criteria, of which 57 patients received RT alone (arm I) and 51 patients received combined chemoradiation (arm II). The risk factors were balanced between both arms (**Table 1**).

**Table 1:** Patients’ characteristics.

Characteristics	Arm I *n*=57 (%)	Arm II *n*=51 (%)	*P*
Age, years			
Median	65	62	0.72
Range	55−72	57−70	
Pathological type			
Endometrioid carcinoma	44 (77.2)	41 (80.4)	0.81
Adenosquamous carcinoma	3 (5.3)	2 (3.9)	1.00
Papillary serous carcinoma	5 (8.7)	6 (11.7)	0.75
Clear cell carcinoma	3 (5.3)	1 (2.0)	0.62
Adenoacanthoma	2 (3.5)	1 (2.0)	1.00
Grade			
1	6 (10.5)	4 (7.8)	0.74
2	23 (40.4)	19 (37.3)	0.84
3	28 (49.1)	28 (54.9)	0.57
FIGO			
IA	8 (14.0)	9 (17.6)	0.79
IB	18 (31.6)	14 (27.4)	0.68
II	31 (54.4)	28 (55.0)	1.00
Lymphovascular space invasion	9 (15.9)	13 (25.5)	0.24

The median age was 65 and 62 years in arm I and II, respectively. Endometrioid carcinoma was the most common pathological type, rate of histologic grade 3 was high in both arms (49% and 54.9%), and most patients were at stage II (54.4% and 55%). Apart from alopecia that occurred in all patients, rates of hematologic toxicities were higher with grade 3 neutropenia and thrombocytopenia in 9.8% and 6% of the patients in arm I and arm II, respectively (**Table 2**). No patients developed febrile neutropenia. No reports of hospitalization were observed during the therapy, and majority of the cycles (91%) were delivered without delay. No treatment-related deaths were reported.

**Table 2:** Chemotherapy related toxicity.

Toxicity	Grade, *n* (%)			
	1	2	3	4
Anemia	4 (7.8)	7 (13.7)	2 (3.9)	0
Neutropenia	5 (9.8)	13 (25.5)	5 (9.8)	0
Thombocytopenia	4 (7.8)	11 (21.6)	3 (6.0)	0
Hypersensitivity	2 (3.9)	0	0	0
Neuropathy	8 (15.7)	6 (11.8)	1 (2.0)	0
Alopecia	0	51 (100)	0	0
Nausea and vomiting	10 (19.6)	2 (3.9)	0	0

After a median follow-up period of 48 months, the relapse rate was higher in patients treated with radiotherapy alone than in those with the combined treatment (22.7% *vs.* 9.8%) (**Table 3**).

**Table 3:** Pattern of relapse.

Site of relapse	Arm I, *n* (%)	Arm II, *n* (%)	*P*
Local	4 (7)	2 (3.9)	0.68
Distant	6 (10.5)	1 (2)	0.28
Both local and distant	3 (5.2)	2 (3.9)	1.0

**Figure 1:**
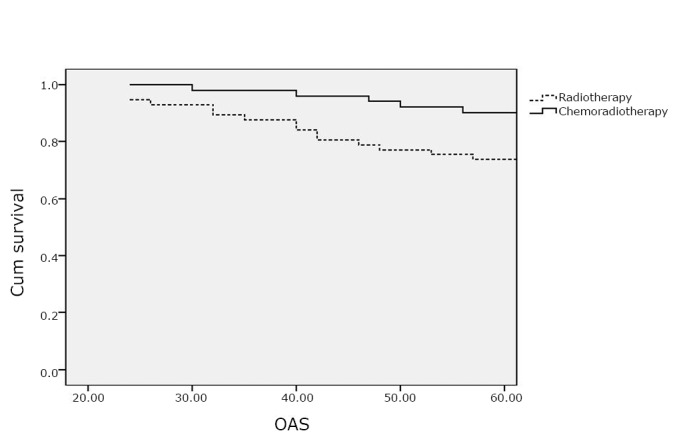
Overall survival (OAS).

**Figure 2:**
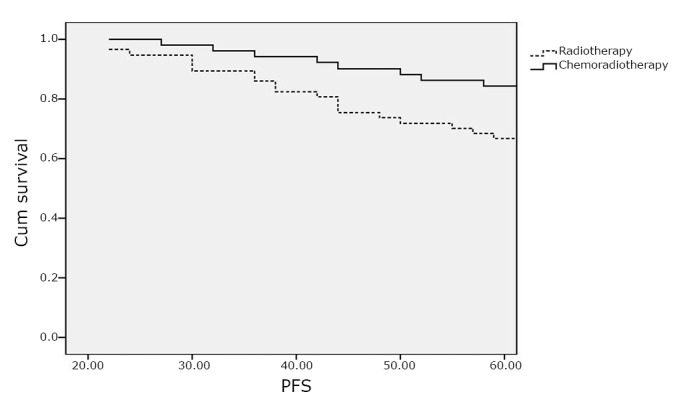
Progression-free survival (PFS).

In arm I, the OAS rate was 73.7% compared with 90.2% in arm II (**Figure 1**), whereas the PFS rates were 66.7% and 84.3% in arm I and arm II, respectively (**Figure 2**). The patients in arm II have significantly improved OAS and PFS rates than those in arm I (*P*=0.02 and 0.03).

## Discussion

Optimal adjuvant therapy in relation with high-risk endometrial cancer is poorly defined. Randomized clinical trials and observations comparing adjuvant pelvic RT with brachytherapy showed significant reduction of the risk of locoregional relapse with pelvic irradiation. But these methods showed no clear trend towards the prevention of distant metastases or the improvement of overall survival^[^^4^^,^^5^^,^^15^^]^.

Chemotherapy has a function in the management of advanced and recurrent endometrial cancer, but that of optimal chemotherapy has not reached a consensus. The combination of cisplatin plus doxorubicin was commonly used for optimal chemotherapy^[^^16^^]^.

The use of paclitaxel in patients with endometrial cancer has attracted attention because of its success in treating ovarian and breast cancers. When paclitaxel was used as a single agent in an advanced or recurrent case, the response rates were 36%-43%, and the activity was demonstrated in the truly platinum-resistant patients^[^^17^^,^^18^^]^. The combination of paclitaxel with platinum analogue was proven to be a more effective regimen by many studies^[^^19^^,^^20^^,^^21^^]^.

In the present study, chemotherapy was associated with an acceptable rate of toxicity. The most common toxicity was hematologic, which coincided with that reported by Michener et al.^[^^22^^]^

All patients had alopecia, which was similar to the observation by Hoskins et al.^[^^19^^]^. The incidence of grade 3 hematologic toxicity was 21.7% with no grade 4 toxicity compared with the recorded grade 3-4 toxicity at 27% by Lupe et al.^[^^23^^]^ Thus, this higher rate can be explained by the increased number of cycles used in their study (6 cycles).

Fader et al.^[^^24^^]^ mentioned that the addition of platinum and taxane to adjuvant RT was associated with the decreased risk of relapse, which is similar to our findings. On the other hand, Kuoppala et al.^[^^25^^]^ found that adjuvant chemotherapy with cisplatin, epirubicin, cyclophosphamide, and RT failed to lower the recurrence rate. Creutzberg et al.^[^^5^^]^ reported a relapse rate of 14% among patients who did not receive radiotherapy, whereas the relapse rate was 22.7% in the radiotherapy group in the present study. These results can be explained by the difference in the inclusion criteria, wherein their patients were stage I and were mostly grades I and II.

The 5-year OAS rates were significantly higher (*P*=0.02) in arm II (90.2% *vs.* 73.7%), whereas the PFS rates were 66.7% and 84.3% in arm I and arm II, respectively (*P*=0.03). These findings were comparable to those reported by Susumu et al.^[^^26^^]^ and Hogberg et al.^[^^27^^]^

The differences in the report of 5-year OAS (85%) by Creutzberg et al.^[^^5^^]^ could be due to that all patients in their study were stage I. In addition, Keys et al.^[^^4^^]^ also reported a 4-year OAS rate of 86% in the ‘No adjuvant treatment’ group because their patients were in the intermediate risk group compared with our patients who were in the high-risk group.

## Conclusions

Adjuvant chemoradiation with paclitaxel and carboplatin improved the survival rates and decreased the recurrence rates in patients with high-risk stage I-II endometrial carcinoma. Chemotherapy was associated with an acceptable rate of toxicity. However, a prospective study with a larger number of patients is needed to define a proper standard of adjuvant treatment for high-risk stage I-II endometrial carcinoma.
